# Management of cervical ectopic pregnancy after unsuccesful methotrexate treatment

**Published:** 2014-04

**Authors:** Siniša Šijanović, Domagoj Vidosavljević, Zlatko Topolovec, Andrea Milostić-Srb, Milanka Mrčela

**Affiliations:** 1 *Cathedra for Gynaecology and Obstetrics, JJ. Strossmeyer University School of Medicine Osijek, Croatia.*; 2 *Institute for pathology and forensic medicine, KBC Osijek, Osijek, Croatia.*

**Keywords:** *Cervical pregnancy*, *Ectopic pregnancy*, *Methotrexate*, *Hysteroscopy*

## Abstract

**Background:** Cervical pregnancy is rare and dangerous form of ectopic pregnancy which can be treated surgically or conservatively. Methotrexate is reasonable conservative option with high efficiency and acceptable level of side effects. Aim of this paper is to present possible treatment option in case of methotrexate failure, still keeping the postulates of minimal invasive surgery.

**Case: **We describe a case of cervical ectopic pregnancy in nulliparous female that was unsuccessfully treated with single dose, local, ultrasound guided intraamniotic methotrexate. Due to vaginal bleeding caused by remaining products of conception a hysteroscopic resection was performed.

**Conclusion:** Despite the problems that can occur in methotrexate treatment, it is still by far, cheapest and most effective treatment of cervical pregnancies. If necessary, procedure can be combined with other minimal invasive surgical procedures leading to satisfactory results. Hysteroscopic resection has enabled us to remove the product of conception from cervix making the minimal damage to the local tissue, thus preserving fertility.

## Introduction

Cervical pregnancy represents a rare but life-threatening type of ectopic pregnancy. Incidence of cervical pregnancy varies between 1:1000 and 1:18000 pregnancies, and it was first described by Rubin (1). In recent years, number of cervical pregnancies rises due to increasing number of in vitro fertilizations (2). Although considered rare, they represent major threat due to its risk of major life threatening hemorrhages. Therefore different methods of treatment were used ranging from hysterectomy up to conservative ones (3). Ultrasound (US) has made diagnosis easier and more accurate also broadening the therapeutic possibilities with ultrasound-guided local injections or aspiration (4). 

Main criteria for US diagnosis of cervical pregnancies were given by Hofmann *et al* and they consist of: no evidence of intrauterine pregnancy, hourglass shape of uterus, cervical ballooning, presence of placental tissue or gestational sac within the cervical canal and closed internal os (5). Treatment can include: dilatation and curettage with intracervical tamponade, angiographic embolisation, cervical cerclage, ligature of uterine arteries or local hysteroscopic endocervical resection of gestational sac with local use of different substances. Different authors describe use of potassium chloride or methotrexate (MTX) intraamniotic or systemic (6, 7). Jeong *et al* described combined method of hysteroscopic management of cervical pregnancy, along with intrauterine irrigation with hydrogen peroxide (H_2_O_2_) (8). Monteagudo *et al*, Yazici *et al*, Cepni *et al,* and Hassiakos *et al* suggest transvaginal, ultrasound with single dose MTX as good non-surgical therapeutic option (3, 6, 9, 10). 

Our experiences with use of MTXin similar cases have been favorable. In recently published case of heterotopic pregnancies, by having administered MTX directly in gestational sac, we managed to eliminate cervical pregnancy without harming intrauterine pregnancy and with good perinatal outcome (11). Jeong *et al *have reported intraamniotic use of MTXmethotrexate in case of triplet cervical pregnancy (12). Aim of this report is to present possible treatment option in case of MTX therapy failure, still keeping the postulates of minimal invasive gynecology.

## Case report

A 31-year-old female, gravida 4, para 0, was admitted to the Clinic for Gynecology, University Hospital Osijek, complaining of mild vaginal bleeding, with last menstrual period late for 11 days. She had previously been of good health, with no significant medical or surgical history. Her pregnancy was achieved spontaneously. Her gynecological history is consisted of 3 spontaneous miscarriages, which in two cases required dilatation and curettage. During her physical and gynecological examination patient was haemodinamically stable and afebrile. Abdominal palpation showed no signs of guarding. Pelvic examination was normal, except of small blood clot protruding from lower lip of cervix, approximately on 7 O’clock position. Cervix itself was closed, uterus and adnexis were of normal size, and there was no sign of cervical “ballooning”.

**Figure 1:**
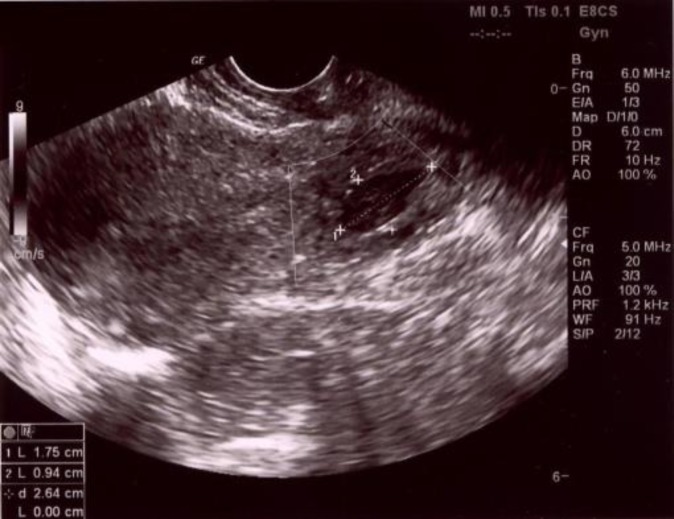
Ultrasonic picture of cervical pregnancy

Transvaginal ultrasound revealed empty uterine cavity, gestational sac within the cervical canal with trophoblastic ring and embryonic echo measuring CRL 3.4 mm (Figure 1). Serum beta human chorionic gonadotropine (βHCG) was measured 16553.5 IU/L, hemoglobin was slightly reduced- 105 g/L, with all other blood and coagulation parameters normal. After admittance different therapeutic options were discussed, but the decision to use locally administered single dose MTX was made, in order to minimize possible effect on future fertile capability of the patient. She received single dose of 50 mg MTX directly by ultrasound guided injection into the gestational sac. After administration of MTX continuous monitoring of her blood level of βHCG was done. From initial 16553.5 IU/L βHCG started decreasing to the 6916.9 IU/L on the 4^th^ day, and transvaginal ultrasound showed collapsed gestational sac with small and irregularly shaped embryonic echo. 

Further serum βHCG monitoring continued with declining values, dropping to 2565.8 IU/L on 8^th^ day and 1083 IU/L on 16^th^ day. Since there was minimal bleeding without other symptoms patient was discharged on the 8^th^ day on home care with instructions to report every 7 days for βHCG serum level control and with possibility to be admitted in case of severe vaginal bleeding or other symptoms. Patient reported to the hospital on 30^th^ day after MTX administration with βHCG level dropping to 162.7 IU/L with minimal vaginal bleeding still present. Decision was then made for continuation of expectative approach.

**Figure 2:**
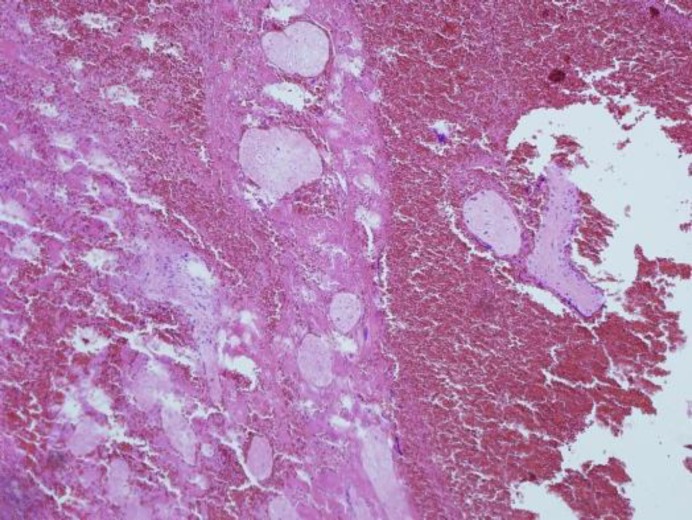
Pathohystologic picture showing products of conception (HE 100x)

Patient reported a week later complaining of moderate vaginal bleeding. Additional βHCG level was made presenting a fall to the level of 77.1 IU/L. Vaginal examination in specula showed residual products of conception in cervix that started bleeding profusely during the examination. Temporarily, a Foley catheter was placed into cervical canal to secure hemostasis. Different procedures were considered but we optioned for hysteroscopy, as least aggressive procedure that enables accurate diagnostics and more treatment options. Products of conception were discovered in posterior lip of the cervix and removed. Pathohistologic diagnosis confirmed existence of products of conceptions (Figure 2). 

Monopolar rollerbal was used to make hemostasis and Fibrillar net was placed in cervical canal to secure hemostasis. Patient was discharged three days later with βHCG level of 1.7 IU/L with no further abnormal bleeding or other symptoms.

## Discussion

The cause of cervical ectopic pregnancies remains unknown. The incidence is less than 1% of all ectopic pregnancies, ranging from 1 in 1000 to 1 in 18000 live births (13). According to literature an increase of cervical pregnancies was noticed after dilatation and curettage due to damage cervical canal (14). Several studies have suggested an increase in incidence of cervical pregnancy in women undergoing in vitro fertilization, often combined as heterotopic pregnancies (15). 

Usually, clinical symptoms of cervical pregnancy are very scarce: mild vaginal bleeding in amenorrhoic patient is dominant, with feeling of abdominal discomfort. Sometimes, during vaginal examination an implantation site with products of conception can be seen, notable as cervical ballooning, but US examination is necessary for confirmation of the diagnosis. However, some points in patients’ medical history are very important and should not be missed. All kinds of manipulations and procedures on cervix are considered as potential causal factor for cervical pregnancies e.g. dilatation, curettage, hysteroscopy etc.

According to the many reported cases, local administration of MTX was successfully used in the management of monofetal or multifetal cervical pregnancies. The success rate of MTX treatment in cervical pregnancy was reported as high as 81.3% (4). Several publications describe successful intra-amniotic MTX administration in monofetal or multifetal cervical pregnancies as a single approach or combined with adjuvant conservative methods (3, 6, 9-12). MTX combined with such methods has a success rate of almost 90%. Systemic application of MTX in multiple doses has been described as one of the methods in treatment of cervical pregnancy, recommending its use in cases of low gestational age fetuses and in the absence of fetal viability (7).

Question arises about 10-18.7% of cervical pregnancies that do not respond well to MTX treatment, and some authors published reports regarding this issue. In such cases, authors report cervical evacuation/ dilatation and curettage, with or without balloon tamponade as first aid measure (16, 17). The usual MTX dose is 1-1.5 mg/ per kg of body weight with a possible risk of systemic adverse effects such as thrombocytopenia, leucopenia, elevated serum liver enzymes, fever and gastrointestinal symptoms (18). Methotrexate treatment is by far the cheapest procedure for cases of cervical ectopic pregnancies, as well as for other cases of intact ectopic pregnancies (without acute complications). Problem arises when MTX makes only partial success.

This case report shows that viable ectopic pregnancy could successfully be resolved with combination of local conservative MTX therapy and local surgical therapy- primarily hysteroscopic resection of products of conception, even in cases when conservative therapy fails. Matteo *et al* have described combined medical-hysteroscopic conservative treatment of viable pregnancy (7). In similar case, Tinelli *et al* have been forced to perform urgent vaginal ligation of the cervical branches of the uterine arteries, suction curettage, dilatation and curettage and insertion of an intrauterine sterile tampon after unsuccesfull use of MTX, followed by massive haemorrhage (17). Grimbizis *et al* have described use of dilatation and curettage along with intracervical tamponade following MTX use in five cases (16). Moon *et al* have described use of Tuohy needle for instillation of local MTX after failure of systemic use of MTX (19). 

In this case we used a combination of ultrasound guided- single dose local intraamniotic injection of 50 mg MTX and 37 days later a hysteroscopic procedure- resection and hemostasis with success at the end; although clinical response to MTX was modest. Expectative approach with close follow up was needed in this case to preserve fertility in patient with burdened medical history, respecting all postulates of minimally invasive gynecology. The gynecologist managing cervical pregnancy should be aware of possible side effects of MTX when considering treatment options. The US guided single dose, intra-amniotic MTX injection has proven successful in the treatment of cervical pregnancy, but the treatment options should vary according to presence of active hemorrhaging, fetal gestational age, vital embryo presence, patient’s desire to maintain fertility and experience of the medical team in using MTX. 

All of the cervical manipulations (dilatation, electrocauterisation) may cause cervical ectopic pregnancies, so even in this case, where hysteroscopy has been useful; it can be a reason for future cervical pregnancy in the perspective. Therefore, any future pregnancy should be kept under suspicion for possible cervical ectopic pregnancy. This method is worth of consideration in similar cases.
